# Human biomonitoring without in-person interaction: public health engagements during the COVID-19 pandemic and future implications

**DOI:** 10.1186/s12874-024-02165-x

**Published:** 2024-02-28

**Authors:** Alyssa J. Mattson, Jiali Yu, Elizabeth M. Miller, Michael Schueller, Michael Pentella, Susie Y. Dai

**Affiliations:** 1 State Hygienic Laboratory, University of Iowa, Iowa City, IA USA; 2 Systems and Synthetic Biology Innovation Hub, Texas A&M University, College Station, TX USA; 3 Department of Plant Pathology and Microbiology, Texas A&M University, College Station, TX USA; 4 Department of Epidemiology, University of Iowa, Iowa City, IA USA; 5 Department of Occupational and Environmental Health, University of Iowa, Iowa City, IA USA

**Keywords:** Biomonitoring, Public health surveillance, Virtual recruitment

## Abstract

**Background:**

Public health initiatives, including human biomonitoring, have been impacted by unique challenges since the onset of the COVID-19 pandemic, compounding a decades-long trend of declining public participation. To combat low public participation rates, public health professionals often employ extensive engagement approaches including in-person interactions related to enrollment and sampling, success of which is an essential component of a statistically defensible study. The onset of the COVID-19 pandemic challenged public health programs to diversify engagement and sampling approaches, limiting direct interactions for the health and safety of the population. This study explores biomonitoring recruitment strategies through non-contact mechanisms and evaluate the application feasibility for population-based studies.

**Methods:**

The Iowa Biomonitoring Program at the State Hygienic Laboratory developed a human biomonitoring study that utilized a multifaceted, distance-based approach. Traditional techniques, such as mailed recruitment invitations and phone-based discussions, were coupled with internet-based surveys and self-collected, shipped urine and water samples. Participation rates were evaluated by employing different mailing methods, and the demographics of enrolled participants were examined.

**Results:**

This non-human contact approach achieved a nearly 14% participation rate among a rural population, well above our target rates. Our improved mailing strategy for targeting initially unresponsive participants yielded a significantly increase in the participation rates. The respondents were predominantly individuals with educational attainment of at least high school level. Among all the eligible participants, 83% submitted self-collected samples, a rate comparable to the National Health and Nutrition Examination Survey which involved in-person interviews.

**Conclusions:**

The practice of engaging a rural population during the COVID-19 pandemic by transitioning from face-to-face interactions to a combination of mailing and internet-based approaches resulted in higher-than-expected participant recruitment and sample collection rates. Given the declining trend in the response rates for population-based survey studies, our results suggest conducting human biomonitoring without direct human interaction is feasible, which provides further opportunity to improve response rates and the relevance and reach of public health initiatives.

**Supplementary Information:**

The online version contains supplementary material available at 10.1186/s12874-024-02165-x.

## Introduction

Human biomonitoring (HBM) is the measurement of environmental chemicals and their metabolites in human tissues to determine rates of potentially harmful exposures. HBM is a public health initiative that often involves population-based surveillance and relies on the collection of human specimens. Analytical tests determine concentration or presence of chemicals to evaluate human exposure to environmental substances or disease agents in a population [1].

The onset of the COVID-19 pandemic caused significant challenges for public health outreach programs which involve direct human contact. The pandemic made it important to explore alternative means to implementing public health initiatives where minimal in-person interactions are required. The utilization of resources that can be distributed without direct contact (i.e., mail and internet-based exchanges) can potentially maintain or enhance public health activities, not only during challenging times, such as the COVID-19 pandemic, but also in broader applications where extensive or distanced subject recruitment and engagement benefit public health.

Thus, the COVID-19 pandemic served as an impetus to redefine public health engagement methods. HBM programs were faced with a distinct challenge of limiting in-person interaction while obtaining adequate response rates and ensuring the quality of sampling and data collection. A comprehensive survey of minimally invasive collections for population-based research was summarized by Lindau et al. [2]. To some extent, self-administrated collection methods and sample shipment can be used to obtain non-invasive biological samples (e.g. urine, saliva, hair, nails, etc.). For example, Weir has evaluated the quality of studies relying on the recruited participants to collect the biological samples [3]. This study suggested that the self-administrated sample collection could be comparable to the trained interviewers’ collection. Those previous studies demonstrated the feasibility of performing the HBM project without direct in-person interaction.

This study evaluates conducting HBM without direct in-person interaction amid the COVID-19 pandemic and compares its utility with public health measures using in-person interviews and sample collection approaches. The study has been regarded as a public health activity, thus was exempted from the Institutional Review Board (IRB) review as determined by the institutional IRB board. The HBM program targeted rural residents that use a private water well as their primary household drinking water source in Iowa, a state in the midwestern United States. Engaging a rural population utilizing private water wells presents unique challenges. The private well population is considered poorly characterized, with states and individual counties lacking comprehensive data on the geographical locations of private wells [4]. In Iowa, a database called the Private Well Tracking System (PWTS) serves as one of the most robust repositories of private wells in the state and served as the basis for our engagement efforts. Randomly selected participants were contacted by mail with an invitation to enroll. Subsequent engagement platforms included internet-based eligibility screening, questionnaire administration, and video education, as well as self-administrated sample collection shipped directly to the laboratory. Telephone support was available to any participants lacking internet access or capability. Recruitment and sample collection rates were compared with data from the Centers for Disease Control and Prevention (CDC) National Health and Nutrition Examination Survey (NHANES) program. This study highlights the feasibility of conducting HBM or similar public health initiatives without in-person interaction and presents a legitimate and convenient model for future HBM studies with cost-saving potential.

## Methods

### Subject selection

Private well data were sourced for 11 counties spanning the state of Iowa (Boone, Buchanan, Cerro Gordo, Clinton, Greene, Hancock, Hardin, Johnson, Monona, Woodbury, and Wright) using the PWTS administered by the Iowa Department of Natural Resources [5]. Data included site locations, construction logs, and water testing details, as available. Private water wells are outside the jurisdiction of the Safe Drinking Water Act (SDWA), and to date, there is no comprehensive database that completely inventories all private wells used for drinking water in Iowa [6]. Data is primarily input by licensed well drillers and county public health officials at the time of well installation, structural assessment, repair, plugging, or water testing. For this project, records in PWTS that had not been updated with activity since 2002 were excluded. Private well owners were selected through stratified random sampling from each county as independent subgroups. Each selected household was verified through the State of Iowa Assessors website. The compiled recruitment data was managed within a custom-built REDCap (Research Electric Data Capture) database, a secure web platform for building and managing online databases and surveys. The platform facilitated data collection, engagement tracking, issuance of incentive payments, and long-term storage of de-identified samples [7].

### Subject recruitment

Individual households were contacted by mail with an invitation to complete an internet or phone-based eligibility screener to enroll in the study. The recruitment mailing schedule is shown in Table 1. The data was consolidated from August 30, 2021 to May 23, 2022. Recruitment efforts included two phases: the first a gradually increasing rate in the number of households engaged and the second a steady effort at maximum capacity (Table 1). Phase I mailings were distributed to 682 participants in nine batches, each two weeks apart. Phase II mailings went to 3005 households in eleven batches, each two weeks apart. Recruitment and engagement techniques were evaluated and adjusted throughout Phase I. The first two batches in Phase I reached a total of 66 potential participants. The program sent the initial invitation letter with two follow-up letters to non-respondents: one USPS standard envelope and one UPS Express envelope. Batches three and four were sent to 77 potential participants and mailings were adjusted to include only a single follow-up letter in a full-size envelope to non-respondents. Additional tactics to promote engagement included: (1) homeowner verification through the State of Iowa Assessors website, (2) simplification of the enrollment steps in the invitation letter, and (3) insertion of an ‘enroll today’ postcard with “at-a-glance” information and enrollment instructions (supplementary material). The Iowa Biomonitoring Program sent recruitment mailings to 3687 participants within the targeted eleven counties between August 30, 2021 and May 23, 2022. Only one adult from each household was allowed to participate, but the selection of the participating adult was deferred to the household to allow for randomization of male and female enrollees. The University of Iowa IRB review board deemed an IRB was unnecessary as the nature of the study is public health activity. Thus the IRB was waived by the official IRB board. However, IRB standards were followed as best practice, including use of an informed consent letter to inform participants of study objectives, ethics, and privacy. The study does not include minors.

### Engagement methods

Enrollment in the program occurred via a simple eligibility form conducted either through an internet-based REDCap survey or over the phone with program staff. After screening, eligible participants provided basic contact information, such as a phone number and email address, giving the program additional means of communication. Program staff followed-up with enrollees by phone to discuss the tasks associated with study participation. Following enrollment, the program utilized several methods for distance-based engagement with participants. A website was developed to provide comprehensive program details to interested participants, including a program overview and objectives, information on target chemicals, instructional videos for sample collection and shipping, and other informative resources. Sampling kits were shipped to each enrollee, which included five primary components: a urine collection kit, a water collection kit, a questionnaire (supplementary material) available either through an internet-based REDCap survey or in print, detailed instructional pamphlet, and shipping materials with a pre-paid return shipping label for UPS overnight ground delivery. The instructional pamphlets for both water and urine sampling were provided (supplementary material) in the shipping packet along with a ‘make a plan’ postcard to help participants plan their activities (supplementary material). The full instruction was also on-line at the program website [8]. For the questionnaire, questions were asked in nine categories, including demographics, household water supply, residential information, food intake in the past 30 days, overall health, work experience, pesticide use, personal care product use, and smoking habits. We expected it may take up to 30 min for the participant to finish the questionnaire. Participants were expected to submit a water sample collected in six bottles (four 250 mL amber glass bottles for collecting organics, one opaque 100 mL plastic bottle with nitric acid for metals, one dark brown plastic bottle with ethylenediaminetetraacetic acid for arsenic speciation), a urine sample in one urine collection cup (a 150 mL clinical urine collection cup), and a completed questionnaire within two weeks. A reminder was sent by mail if the questionnaire was not received at the lab with the samples. Upon completion of the questionnaire and sampling, participants shipped their samples back to the laboratory. Participants received up to $50 in gift cards by mail for completion of the various tasks ($15 for submission of the questionnaire and $35 for submission of both urine and water samples). All reportable testing results were sent to the participant by mail. Program resources were made available on the program website and participants were invited to contact staff by phone or email with questions. Additional consultation resources regarding well water quality and environmental exposures were available through program partners in the Iowa Poison Control Center and the individual County Environmental Health Departments.

### Statistical analysis

Participants who responded to either the first or second mailings as eligible to enroll were encoded into binary data with eligible as ‘1’ and no response as ‘0’. One-hot encoding was treated to the categorical variable of the four different mailing methods, including address verification, second mailing, simplified letter, and insert card. We also considered the potential influence of various months through the year on response outcomes; thus the mailing time was recoded to categorical data representing different months. To determine if mailing time or sending a second mail was associated with more responses, a logistic regression using a generalized linear model was applied. For the phase I recruitment where additional tactics were applied to promote engagement, a Pearson’s Chi-Square contingency analysis was used to determine the differences between responses to different mailing methods. In addition, we emphasized the necessity of verifying address, by comparing the response rates before and after address verification in phase I recruitment. The statistical difference of their response rates was determined by Welch’s t-test. Statistical significance was considered with a p-value < 0.05 threshold. All collected data were analyzed in R using ‘stats’ package.

### Data source

We collected the publicly available response rate data from CDC NHANES cycles from 1971 to pre-pandemic [9]. Due to the low response rate since 2015, the response rates in the later cycles were adjusted for the screener response rate following NHANES instructions. Demographic data and dietary data were from NHANES 2015–2016 cycle because ‘well water’ was an answer option in that cycle. After 2016, this option was eliminated. We thus extracted the well users’ demographic information only from the 2015–2016 cycle [10]. Participants were filtered to only include those who reported drinking tap water from well or rain cistern. The demographic data of the total population from Iowa were collected from 2021 one-year American Community Survey (ACS) from the Iowa State Data Center [11].

## Results and discussion

### Participant recruitment and sample collection rates

The Iowa Biomonitoring Program contained two phases for recruitment; Phase I included nine batch mailings over 18 weeks and Phase II included eleven batches of mailings over 22 weeks. Several modifications were made to the engagement process during Phase I of this study to improve response rates as the rate of distributions gradually increased. The first two batches were distributed to a total of 66 households and elicited 4 enrollments (6%). Subsequent recruitment efforts during Phase I reached a total of 616 additional households, resulting in 90 enrollments. Enrollment rates for Phase I reached 13.78% (Table 1). Phase II of recruitment mailings proceeded at a rate of 275 households per batch through batch 18, at which time valid well records in one of the target counties were exhausted. Phase II recruitment mailings reached a total of 3005 households and garnered 417 enrollments, maintaining a comparable 13.88% response rate.

Enrolled participants were provided materials to self-collect urine and water samples and complete an accompanying questionnaire from their home. Rates of samples submission between the first batch mailing on August 30, 2021 and the conclusion of our data collection period on August 30, 2022, accounted for 425 submissions from the 511 enrollees (83%). Details of enrollment and subsequent project completion are summarized in Table 1.

**Table 1:** Rates of participant enrollment and sample collection over two project phases

Project Phase	Mailing Batch	Mailing Distributed	Enrollment Rate	Sample Submission Rate
Phase I (Batches 1–9)	1	33	3	3
	2	33	1	1
	3	33	8	6
	4	44	7	7
	5	44	3	3
	6	55	9	9
	7	99	12	8
	8	165	25	23
	9	176	26	21
Phase II (Batches 10–20)	10	275	46	41
	11	275	54	51
	12	275	37	32
	13	275	39	33
	14	275	33	30
	15	275	41	32
	16	275	28	24
	17	275	41	25
	18	265	33	28
	19	270	31	25
	20	270	34	23
	**Total**	**3,687**	**511**	**425**

### Study group demographics

Demographic characteristics of study participants were gathered through a questionnaire completed at the time of sample collection. Questionnaire responses were submitted either through the internet-based REDCap survey portal or on a hard copy that was then input into REDCap by program staff. Results are summarized in Table 2. Of the 511 enrolled participants, 420 completed the questionnaire (82% submission rate). Participants included 215 males (51.2%) and 204 females (48.6%), while one participant did not report gender. Most enrolled individuals were white (98.6%), while four participants did not report. Of the 419 participants that reported their educational degrees, more than 85% of them obtained a degree higher than a high school diploma. 70% of the participants have lived in the household for more than 10 years, and only 4% have lived in the household for less than 2 years. Occupations were widely diverse, with management as the most reported occupation (69, 16.4%). Most of the household incomes ranged between $50,000 to $150,000 (253, 60.2%), while 18 households (4.3%) reported a yearly income less than $25,000.

**Table 2:** Demographic characteristics of enrollees having completed the questionnaire in our study compared to NHANES and Iowa state populations

Characteristic	Category	Result (*n* = 420)	NHANES^a^ (*n* = 596)	Iowa^b^
Sex	Male	215 (51.2%)	327 (54.9%)	50.2%
	Female	204 (48.6%)	269 (45.1%)	49.8%
	Not reported	1 (0.2%)	0	-
Race	White	414 (98.6%)	396 (66.4%)	84.5%
	Black or African American	1 (0.2%)	33 (5.5%)	3.6%
	Hispanic	-	132 (22.1%)	6.6%
	American Indian or Alaska Native	0	-	0.5%
	Native Hawaiian or Other Pacific Islander	0	-	0.2%
	Asian	1 (0.2%)	7 (1.2%)	2.8%
	Not reported	4 (1.10%)	0	-
Education^c^	Less than high school graduate	2 (0.5%)	109 (28.5%)	6.6%
	High school graduate (or equivalent)	49 (11.7%)	98 (25.7%)	30.3%
	Some college, no degree	83 (19.8%)	112 (29.3%)	20.6%
	Associate degree in college (2-year)	85 (20.2%)		12.0%
	Bachelor’s degree in college (4-year)	120 (28.6%)	63 (16.5%)	20.7%
	Master’s degree (example: MA, MS, MEng, EEd)	54 (12.9%)		9.9%
	Doctoral degree (example: PhD, EdD)	10 (2.4%)		
	Professional degree (example: MD, DDS, DVM)	16 (3.8%)		
	Not reported	1 (0.2%)	0	-
Total length of residence	1–2 years	17 (4.0%)		
	3–5 years	53 (12.6%)		
	6–10 years	52 (12.4%)		
	More than 10 years	294 (70.0%)		
	Not reported	4 (1.0%)		
Occupation	Academic Research	1 (0.2%)		
	Architecture and Engineering	10 (2.4%)		
	Arts, Design, Entertainment, Sports, and Media	6 (1.4%)		
	Building and Grounds Cleaning and Maintenance	1 (0.2%)		
	Business and Financial Operations	11 (2.6%)		
	Community and Social Service	7 (1.7%)		
	Computer and Mathematical	6 (1.4%)		
	Construction and Extraction	8 (1.9%)		
	Educational Instruction and Library	40 (9.5%)		
	Farming, Fishing, and Forestry	39 (9.3%)		
	Food Preparation and Serving Related	4 (1.0%)		
	Healthcare Practitioners and Technical	44 (10.5%)		
	Healthcare Support	18 (4.3%)		
	Installation, Maintenance, and Repair	19 (4.5%)		
	Legal	1 (0.2%)		
	Life, Physical, and Social Science	6 (1.4%)		
	Management	69 (16.4%)		
	Office and Administrative Support	28 (6.7%)		
	Other	35 (8.3%)		
	Personal Care and Service	5 (1.2%)		
	Production	17 (4.0%)		
	Protective Service	2 (0.5%)		
	Sales and Related	25 (6.0%)		
	Transportation and Material Moving Occupations	13 (3.1%)		
	Not reported	5 (1.2%)		
Income	Less than $25,000	18 (4.3%)	116 (22.9%)	19.5%
	$25,000–49,999	51 (12.1%)	280 (47.0%)	23.3%
	$50,000–74,999	74 (17.6%)		13.9%
	$75,000–99,999	77 (18.3%)	53 (8.9%)	9.0%
	$100,000–149,999	102 (24.3%)	110 (18.5%)	17.9%
	$150,000 or more	84 (20.0%)		18.9%
	Not reported	14 (3.3%)	32 (5.4%)	-

^a^Participants reporting their primary tap water as ‘well or rain cistern’ in the CDC NHANES 2015–2016

^b^Percentage of the total population in Iowa from 2021 American Community Survey 1-year estimate (*n* = 3,200,517)

^c^Educational attainment from population over 20 years old in the NHANES participants, and over 25 years old in the ACS

To access the characteristics of enrolled participants, demographics from CDC NHANES and census were used as references. We particularly analyzed demographics from NHANES participants who reported tap water resource for ‘well or rain cistern’ in the 2015–2016 cycle as these participants primarily used well water. The overall demographics of the population in the state of Iowa was also compared. Our results showed fewer enrolled participants (0.5%) with low education levels than NHANES participants using private well water (28.5%). With 30.6% of the overall population in Iowa obtaining a bachelor’s degree or higher, our participants reached 47.7%. Our participants also reported a higher annual household income compared to the state population and NHANES participants. Taking together, participants enrolled in our study were relatively more well-educated, residentially stable, and had higher income with professional skills as compared with the NHANES general population study.

### Practice implications

**Fig. 1:**
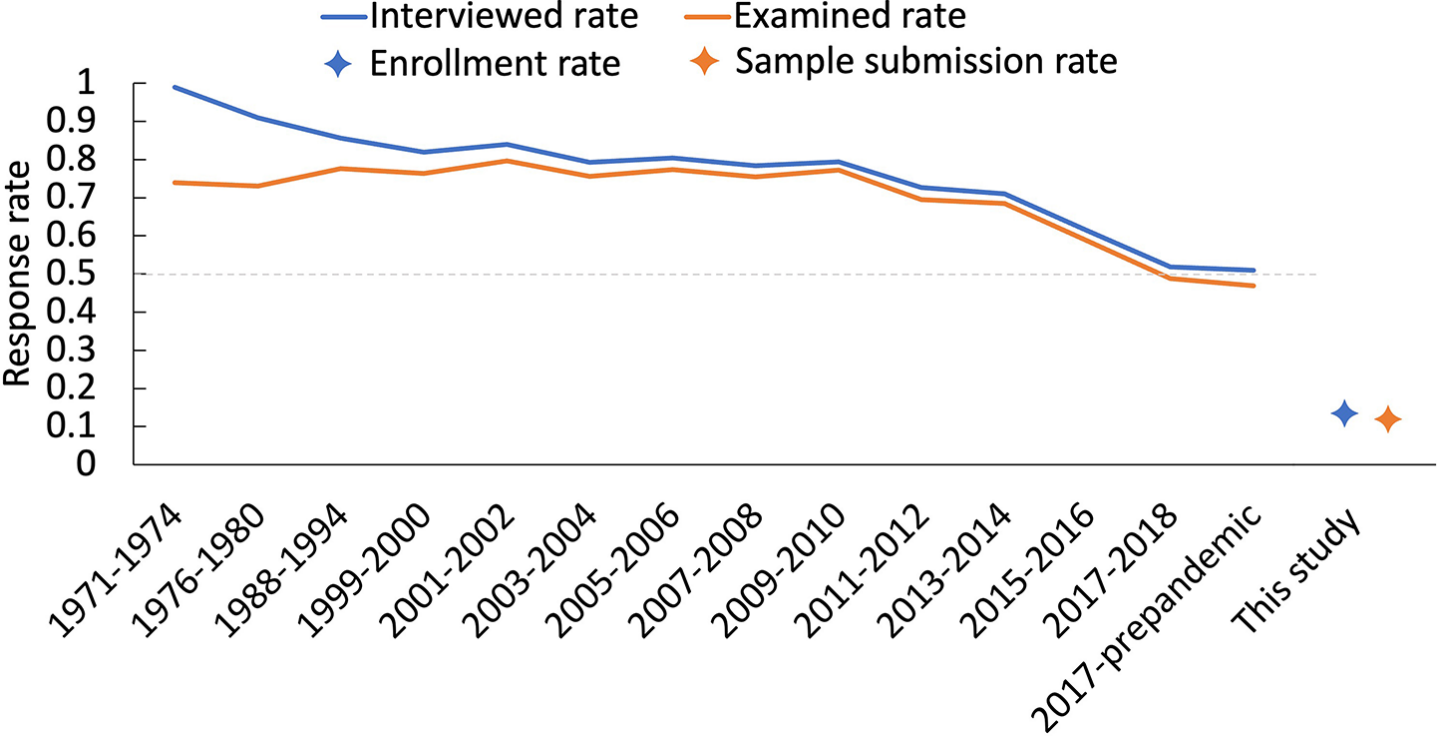
Comparison of CDC NHANES and Iowa Biomonitoring Program rates. Blue and orange lines represent NHANES interviewed and examined rates, respectively. Blue and orange stars represent enrollment and sample collection rates for the Iowa Biomonitoring Program, respectively

The mode of recruitment for the Iowa Biomonitoring Program was entirely based on mailings for initial contact followed by the participant initiating a survey to screen for eligibility in order to complete enrollment. The survey could occur either through an internet-based form or by telephone. As a point of reference, the CDC NHANES interview and examined rates were plotted by date from the time of program initiation through the most recently reported figures in 2018 (CDC 2022) (Fig. 1). When the NHANES program launched in the 1970s, the interview rate was close to 100%, with the examined rate exceeding 70%. However, the interview rate has gradually declined, with a 50% rate in the 2017–2018 program cycle. The Iowa Biomonitoring Program contacted 3687 households by mail with an enrollment rate of 13.86%. Adjustment for ineligible enrollees brings the enrollment rate to 14.50%. The Iowa Biomonitoring Program rate is lower than the NHANES interview rate, but it should be noted that NHANES is a more robustly funded program that combines mailings with in-person solicitations to garner participation, thus, we would expect NHANES to achieve higher rates.

The primary objective when enrolling participants for a HBM study is to gather biological samples for chemical analysis, therefore, the rate of sample submission following enrollment is an important parameter to consider. The sampling model for the Iowa Biomonitoring Program was self-collection of both water and urine samples, which were then shipped back to the laboratory using a pre-paid shipping label. The final sample submission rate from enrolled participants was 83%. This rate is similar to the NHANES program, which reported a 92.3% examined rate of those interviewed.

In this study, we were interested in identifying factors that have the most important impact on influencing an individual’s willingness to participate in the study. The first two mailing cycles of Phase I had a significantly higher rate (45.4%) of returned, undeliverable mail due to incorrect resident information. As a result, our program began conducting verification of current deed holders by address through publicly available data from the State of Iowa Assessors website. The correction of recipient names had a significant impact on the rate of delivered mail, lowering the rate of returned mail to 9% between batches 3–20. The decrease of returned mail did not have a statistical difference in enrollment rates between batches with or without mailing addresses manually verified (p-value = 0.287, Table S1), but did ensure a broader reach within the target population. The most statistically impactful method of improving enrollment rates came with a single follow-up mailing. Of the 511 enrollees, 269 responded to the first mail, while a follow-up letter to non-respondents resulted in an additional 242 enrollments. The second mailing also showed as a significant factor (*p* = 2.2e-16) that is associated with participant responses (Table S2). As such, the combined practice of address verification, sending a follow-up invitation letter in a larger envelope, simplification of the enrollment steps in the invitation letter, and the addition of an insert card with at-a-glance information and enrollment instructions resulted in an increased rate of enrollment (χ² = 85.03, p-value = 0.0004998, Table S3).

**Fig. 2:**
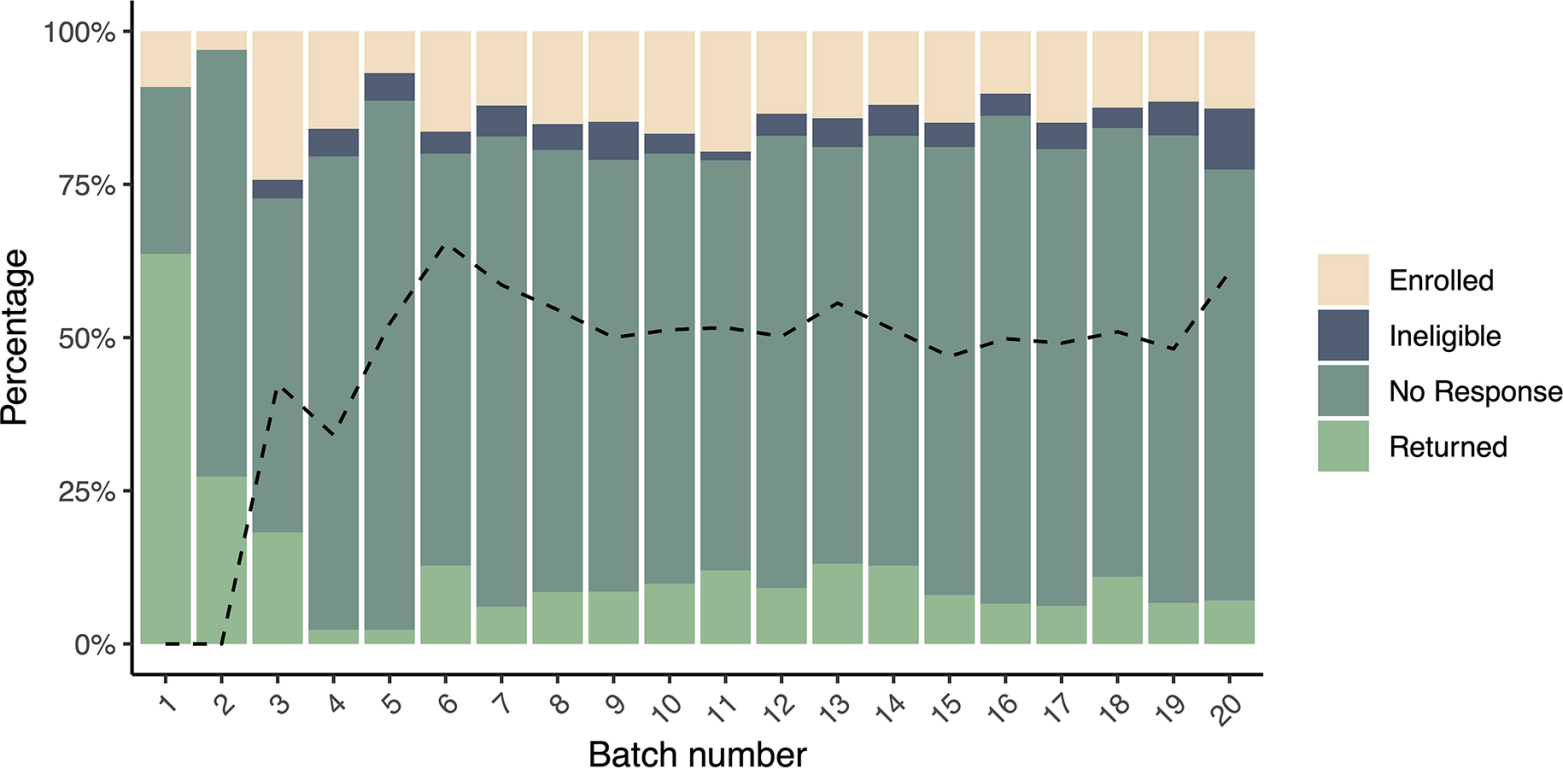
Batch mailing response result composition. Dash line indicated the rate after address corrections

Furthermore, our analysis showed there was no significant difference between selected counties regarding response rates. In summary, our results suggested that sending a follow-up correspondence in a more prominent envelope, as well as ensuring engagement materials are simple, eye-catching, easy to understand, and convenient to act upon, are all promising approaches to increasing responses in biomonitoring recruitment. The response rates to mailings are summarized in Fig. 2.

Additional factors influenced response rates but were not analyzed in this study. The collection of water samples and non-invasive urine specimens serves the purpose of biomonitoring drinking water quality in the rural population. Among different biological fluid samples, urine is a well-established metric to assess exposure risk of environmental toxins [12]. The self-sampling of urine is considered as one of the simplest methods compared to other sampling methods such as dry blood spot or saliva collection, which require more sampling instructions and effort. Our sampling completion rate reached 83.56%, higher than the collection rates (i.e., 71%) from dry blood spot and saliva sampling methods [13]. A study also showed that less educated respondents were less likely to provide saliva samples [14]. The response rate may be influenced if the participants need extra efforts, especially for the population using private well water, who could be less educated (Table 2).

Using incentives could be another factor that influenced the response rates. This study has two types of incentives: monetary (post-completion gift card rewards), and nonmonetary (free water and urine testing results). Previous studies have shown that incentives increased response rates and survey completion rates [15–17]. To increase participation after the pandemic, NHANES 2021–2022 cycle offered a $25 interview incentive to each participant, and the overall incentives for the completion of entire examination ranged from $130 to $175 [18]. As our goal for this study was to reach a high enrollment rate, and to collect as many samples as possible, we provided a $15 gift card upon questionnaire completion and a $35 gift card upon receipt of water and urine samples at the laboratory. Studies showed that pre-incentives increased survey response rates and retention rates, but post-incentives only increase retention rates, not response rates [19]. Both nonmonetary and monetary incentives may increase the response rate of enrollment in our study, however, the gift card rewards are more likely to increase the questionnaire and sample completion rates.

## Perspective

This study aimed to evaluate engagement practices during challenging times, such as the COVID-19 pandemic, when it may not be feasible for public health professionals to directly interact with participants. Additional compounding challenges in public health surveillance programs, including HBM, include limited study resources and the declining rate of public participation in general. Our study suggests that with limited resources, a public health outreach program may successfully leverage various distance-based engagement strategies. The Iowa Biomonitoring Program employed an engagement model that coupled traditional techniques, such as mailed correspondence, with subsequent internet or phone-based interactions to achieve an approximate 14% enrollment rate during the height of the COVID-19 pandemic. Furthermore, the Iowa Biomonitoring Program pursued non-invasive specimens (e.g., urine) to facilitate quality, self-collected sampling by participants, proving to be a viable approach for population-based public health studies.

There are a couple of implications to this study given the ongoing challenges with public health outreach initiatives. First, adequate sample collection and survey responses are necessary to effectively evaluate population exposure characteristics and risks, making initial engagement and enrollment a critical step in the success of the study. In recent decades, the survey field has experienced a steady decline in recruitment rates in population studies. The general population survey started transitioning away from interviewer-administrated modes in the early 2000s [20]. The same trend has been observed in the biomonitoring field, as the CDC NHANES has recorded declining response rates [9]. The decreased response rate has been consistently observed in other population-based studies. For example, a study by Pew Research Center suggested that the telephone survey response rate dropped to 6% around 2018 [21]. However, it has been recognized that low response rates (i.e., 10% or below) may not necessarily invalidate the utility of the survey. Instead, the scientific community has transitioned to a mixed mode of conductive surveys [20]. Second, the target rural population is regarded as challenging for public health measures [4]. Previous studies targeting this population have primarily focused on contaminants such as arsenic, bacteria, and nitrates [22–25].

Nevertheless, this study has several limitations. First, the study did not examine additional practices for participant recruitment, such as telephone solicitation. Second, there is not a control group designed to compare practice improvement. Given the ongoing nature of this study, it is not practical to design a statistical control to maintain a static practice during the biomonitoring recruitment period. Third, the current study does not account for other factors, such as how internet accessibility may or may not impact virtual engagement. Fourth, this study focuses on engagement within a largely white, middle-class, rural population with English as a first language, which may not generally apply to a more diverse, urban population. We expect an evaluation of other tools in the future to enhance recruitment tactics and increase response rates for environmental health studies in similarly characterized populations. Among the future tactics that could be considered for enhanced recruitment would be the use of telehealth technology employed in healthcare systems to reduce in-person interaction. The use of electronic information and telecommunication technologies could support long-distance recruitment efforts much the same as healthcare providers used these tools for clinical health care, patient and professional health-related education, and health administration. The COVID-19 pandemic required that innovative tools be used to communicate with patients and these tools rapidly gained broad acceptance among physicians and insurance providers [26, 27]. Evaluations of this approach showed benefits to include reduced costs, removal of time and demographic barriers and patient convenience [28–30]. This approach provides several convenient, electronic methods of communication including telephone, video, texting, and other internet-based tools. This has been proven to work in the healthcare industry and utilization of these tools should be further studied for use and support in the public health domain, especially when trying to reach a large subset of the population for surveillance studies such as biomonitoring.

## Conclusion

In conclusion, this study highlights the feasibility of conducting a public health surveillance project, such as HBM, with limited resources in a rural population without direct in-person interaction. This approach can serve as a potential model for public health initiatives, especially during pandemics or other situations where human contact poses a risk to health. Our results suggest that using a combination of mailing and internet-based approaches successfully engage participants and may reduce costs associated with face-to-face interactions, thereby improving the efficiency of public health research and surveillance.

### Electronic supplementary material

Below is the link to the electronic supplementary material.

Supplementary Material 1

Supplementary Material 2

Supplementary Material 3

Supplementary Material 4

Supplementary Material 5

## Data Availability

The datasets used and/or analyzed during the current study are available from the corresponding author on reasonable request.

## Abbreviations

ACS: American Community Survey
CDC: Centers for Disease Control and Prevention
HBM: Human biomonitoring
IDNR: Iowa Department of Natural Resources
IRB: Institutional Review Board
NHANES: National Health and Nutrition Examination Survey
PWTS: Private Well Tracking System
REDCap: Research Electric Data Capture
SDWA: Safe Drinking Water Act
