# Supplementary material for: Human biomonitoring without in-person interaction: public health engagements during the COVID-19 pandemic and future implications
Source: BMC Med Res Methodol. 2024 Feb 28;24:53. doi: 10.1186/s12874-024-02165-x (PMC10900566; doi:10.1186/s12874-024-02165-x)
Supplement: Supplementary file 1 — Supplementary Material 1 [file 12874_2024_2165_MOESM1_ESM.docx]

Supplementary Information

**Table S1: Welch's t-test of response rates for batches with and without address verification**

|  | Unverified | Address verified |
| --- | --- | --- |
| Mean | 0.04545455 | 0.14095052 |
| Variance | 0.00413223 | 0.00121267 |
| Observations | 2 | 17 |
| Hypothesized Mean Difference | 0 |  |
| df | 1 |  |
| t Stat | -2.0655567 |  |
| P(T<=t) two-tail | 0.28703403 |  |
| t Critical two-tail | 12.7062047 |  |

**Table S2: First and second mail response rate.**

|  | Outgoing mail | Responded | Withdrew | Deliverable | GLM p-value |
| --- | --- | --- | --- | --- | --- |
| Letter 1 | 3,687 | 269 | 8 | 2,887 | NA |
| Letter 2 | 2,887 | 242 | 11 |  | 2.2e^-16^ |
| Total | 6,574 | 511 | 19 |  |  |

**Table S3: Mailing methods and response rates in the phase I recruitment.**

| Mailing method | Batch | Number of letters | Response (number/rate) | Chi-square test p-value |
| --- | --- | --- | --- | --- |
| Unimproved | 0001 – 0006 | 66 | 3/0.045 | 0.0004998 |
| Address verified, Stamp, First class 2^nd^ mail – Method 1 | 0007 - 0009 | 32 | 7/0.218 |  |
| Method 1 + Simplified letter – Method 2 | 0010 - 0013 | 42 | 7/0.167 |  |
| Method 2 + Insert card – Method 3 | 0014 - 0062 | 513 | 77/0.150 |  |
