# Supplementary material for: Human biomonitoring without in-person interaction: public health engagements during the COVID-19 pandemic and future implications
Source: BMC Med Res Methodol. 2024 Feb 28;24:53. doi: 10.1186/s12874-024-02165-x (PMC10900566; doi:10.1186/s12874-024-02165-x)
Supplement: Supplementary file 2 — Supplementary Material 2 [file 12874_2024_2165_MOESM2_ESM.pdf]

Biomonitoring Questionnaire

Start of Block: Participant Verification

Q1.1 Please enter the Participant ID provided to you by the Biomonitoring Program.

____

Q1.2
Please verify: Are you the person from the household that is providing a urine sample for the project?

○ Yes (1)

○ No (2)

Skip To: End of Survey If Please verify: Are you the person from the household that is providing a urine sample for the pro... = No

End of Block: Participant Verification

Start of Block: Demographics

Q2.1 The following questions ask about you and your background.

Q2.2 What is your sex?

○ Male (1)

○ Female (2)

Q2.3 How many people are currently living at this address?

- 1 (1)
- 2 (3)
- 3 (4)
- 4 (5)
- 5 (6)
- 6 (7)
- 7 (8)
- 8 (9)
- 9 (10)
- 10 (11)
- Other (12) ____

Q2.4 How many children (under 18 years old) live at this address?

- 1 (1)
- 2 (2)
- 3 (3)
- 4 (4)
- 5 (5)
- 6 (6)
- 7 (7)
- 8 (8)
- 9 (9)
- 10 (10)
- Other (11) ____

Q2.5 About how long have you lived at this residence?

- Less than one year (1)
- 1 - 2 years (2)
- 3 - 5 years (3)
- 6 - 10 years (4)
- More than 10 years (5)

Q2.6 Do you consider yourself to be Hispanic, Latino, or of Spanish origin? (Puerto Rican, Cuban/Cuban American, Dominican Republic, Mexican/Mexican American, Central/South American, Other Latin American, Other Hispanic or Latino)

- ○ Yes (1)
- ○ No (2)
- ○ Don't know (3)

Q2.7 What race or races do you consider yourself to be? Please select all that apply.

- ☐ American Indian or Alaska Native (3)
- ☐ Asian (4)
- ☐ Black or Africkan American (5)
- ☐ Native Hawaiian or Other Pacific Islander (6)
- ☐ White (7)

Q2.8 What is the highest grade or level of school you have completed or the highest degree you have received?

- ○ Less than high school graduate (1)
- ○ High school graduate (high school diploma or equivalent including GED) (2)
- ○ Some college, no degree (3)
- ○ Associate degree in college (2-year) (4)
- ○ Bachelor's degree in college (4-year) (5)
- ○ Master's degree (example: MA, MS, MEng, EEd, MBA) (6)
- ○ Doctoral degree (example: PhD, EdD) (7)
- ○ Professional degree (example: MD, DDS, DVM, JD) (8)

End of Block: Demographics

Start of Block: Water

Q3.1 The following questions ask about your household water supply.

Q3.2 Approximately what year was your home constructed?

- ○ Prior to 1950 (1)
- ○ 1950 – 1959 (2)
- ○ 1960 – 1969 (3)
- ○ 1970 – 1979 (4)
- ○ 1980 – 1989 (5)
- ○ 1990 – 1999 (6)
- ○ 2000 – 2009 (7)
- ○ 2010 or after (8)

Q3.3 Approximately when was the **water well** serving your home constructed?

- ○ Prior to 1950 (1)
- ○ 1950 – 1959 (2)
- ○ 1960 – 1969 (3)
- ○ 1970 – 1979 (4)
- ○ 1980 – 1989 (5)
- ○ 1990 – 1999 (6)
- ○ 2000 – 2009 (7)
- ○ 2010 or after (8)
- ○ Don’t know (9)

Q3.4 When was the last time the well was serviced or maintained (for example, structural repairs, pump repair or replacement, etc)

- Less than 6 months (1)
- Between 6 months and 1 year (2)
- Between 1 and 2 years (3)
- More than 2 years (4)
- Don’t know (5)

Q3.5
Have you ever noticed any of the following problems with the quality of your tap water? Select all that apply.

- Abnormal taste (1)
- Abnormal smell (2)
- Abnormal color or cloudiness (3)
- None of these (4)

Q3.6 Have you tested your well water for bacteria (total coliform and/or *E. coli*) or nitrates in the past year?

- Yes (1)
- No (2)
- Don't know (3)

Display This Question:

If Have you tested your well water for bacteria (total coliform and/or E. coli) or nitrates in the p... = Yes

Q3.7 Were any contaminants detected in the testing that you know of?

- ◯ Yes. Please list any that you remember. (1) ____
- ◯ No (2)
- ◯ Don't know (3)

Q3.8 Have you ever shock chlorinated your well for disinfection?

- ◯ Yes (1)
- ◯ No (2)
- ◯ Don't know (3)

Display This Question:

If Have you ever shock chlorinated your well for disinfection? = Yes

Q3.9 When was the last time you shock chlorinated the well?

- ◯ Less than 1 year ago (1)
- ◯ 2 - 3 years ago (2)
- ◯ 4 - 5 years ago (3)
- ◯ More than 5 years ago (4)

Q3.10 Do you use any of the following in-home treatment systems to treat the water entering the residence from the well? (select all that apply)

- [ ] Reverse osmosis (1)
- [ ] Water Softener (10)
- [ ] Charcoal filtration at an individual tap (2)
- [ ] Charcoal filtration under the sink (3)
- [ ] Whole-house charcoal filtration (4)
- [ ] Point of use filter (single-tap filter, refrigerator filter) (5)
- [ ] UV System (11)
- [ ] Other (6) ____
- [ ] No treatment systems (7)

Display This Question:

If Do you use any of the following in-home treatment systems to treat the water entering the residen... = Water Softener

Q3.11 When was the last time the water softener was serviced or maintained?

- ○ Less than 6 months (1)
- ○ Between 6 months and 1 year (2)
- ○ Between 1 and 2 years (3)
- ○ More than 2 years (4)

Display This Question:

If Do you use any of the following in-home treatment systems to treat the water entering the residen... = Reverse osmosis

Q3.12 When was the last time the reverse osmosis system was serviced or maintained (for example, filter replacement)?

- Less than 6 months (1)
- Between 6 months and 1 year (2)
- Between 1 and 2 years (3)
- More than 2 years (4)

Display This Question:

If Do you use any of the following in-home treatment systems to treat the water entering the residen... = Charcoal filtration at an individual tap

Q3.13 When was the last time the charcoal filtration at the tap was serviced or maintained (for example, filter replacement)?

- Less than 6 months (1)
- Between 6 months and 1 year (2)
- Between 1 and 2 years (3)
- More than 2 years (4)

Display This Question:

If Do you use any of the following in-home treatment systems to treat the water entering the residen... = Charcoal filtration under the sink

Q3.14 When was the last time the charcoal filtration under the sink was serviced or maintained (for example, filter replacement)?

- Less than 6 months (1)
- Between 6 months and 1 year (2)
- Between 1 and 2 years (3)
- More than 2 years (4)

Display This Question:

If Do you use any of the following in-home treatment systems to treat the water entering the residen... = Whole-house charcoal filtration

Q3.15 When was the last time the whole-house charcoal filtration system was serviced or maintained (for example, filter replacement)?

- Less than 6 months (1)
- Between 6 months and 1 year (2)
- Between 1 and 2 years (3)
- More than 2 years (4)

Display This Question:

If Do you use any of the following in-home treatment systems to treat the water entering the residen... = Point of use filter (single-tap filter, refrigerator filter)

Q3.16 When was the last time the point of use filter (for example: single-tap filter, refrigerator filter) was serviced or maintained (for example, filter replacement)?

- Less than 6 months (1)
- Between 6 months and 1 year (2)
- Between 1 and 2 years (3)
- More than 2 years (4)

Display This Question:

If Do you use any of the following in-home treatment systems to treat the water entering the residen... = Other

Q3.17 When was the last time the "other" form of water treatment was serviced or maintained (for example, filter replacement)?

- Less than 6 months (1)
- Between 6 months and 1 year (2)
- Between 1 and 2 years (3)
- More than 2 years (4)

Q3.18 Do you use tap water, whether treated or untreated, for any of the following? (select all that apply)

- ▢ Drinking water (1)
- ▢ Cooking (2)
- ▢ Making tea or coffee (3)
- ▢ Bathing (4)
- ▢ Brushing teeth (8)
- ▢ Other (please specify) (7) ____
- ▢ Living in the home but not using any tap water (6)

Display This Question:

If Do you use tap water, whether treated or untreated, for any of the following? (select all that ap... = Drinking water

Q3.19 During the **past 5 days**, including today, on how many days did you drink at least one 8 ounce glass of tap water?

- ○ 0 (1)
- ○ 1 (2)
- ○ 2 (3)
- ○ 3 (4)
- ○ 4 (5)
- ○ All 5 days (6)

Display This Question:

If Do you use tap water, whether treated or untreated, for any of the following? (select all that ap... = Cooking

Q3.20 During the **past 5 days**, including today, on how many days did you use water in preparing a meal using tap water?

- ○ 0 (1)
- ○ 1 (2)
- ○ 2 (3)
- ○ 3 (4)
- ○ 4 (5)
- ○ All 5 days (6)

Display This Question:

If Do you use tap water, whether treated or untreated, for any of the following? (select all that ap... = Making tea or coffee

Q3.21 During the **past 5 days**, including today, on how many days did you consume at least one 8 ounce cup of tea or coffee using tap water?

- ○ 0 (1)
- ○ 1 (2)
- ○ 2 (3)
- ○ 3 (4)
- ○ 4 (5)
- ○ All 5 days (6)

Display This Question:

If Do you use tap water, whether treated or untreated, for any of the following? (select all that ap... = Bathing

Q3.22 During the **past 5 days**, including today, on how many days did you take a shower or bath using tap water?

- ○ 0 (1)
- ○ 1 (2)
- ○ 2 (3)
- ○ 3 (4)
- ○ 4 (5)
- ○ All 5 days (6)

Display This Question:

If Do you use tap water, whether treated or untreated, for any of the following? (select all that ap... = Brushing teeth

Q3.23 During the **past 5 days**, including today, on how many days did you brush your teeth using tap water?

- ○ 0 (1)
- ○ 1 (2)
- ○ 2 (3)
- ○ 3 (4)
- ○ 4 (5)
- ○ All 5 days (6)

End of Block: Water

Start of Block: Area

Q4.1 The following questions ask about the area around your residence.

Q4.2 Approximately how far is your well from your septic tank (or human waste lagoon)?

- ◯ Less than 50 feet (1)
- ◯ 50 - 100 feet (2)
- ◯ 100 - 200 feet (3)
- ◯ Greater than 200 feet (4)
- ◯ Don't know (5)

Q4.3 Do you own or work on a farm?

- ◯ Yes (1)
- ◯ No (2)

Display This Question:

If Do you own or work on a farm? = Yes

Q4.4 What are the major income producing crops and animals you are currently raising on the farm? (Select all that apply)

- ☐ Beef cattle (1)
- ☐ Dairy cattle (2)
- ☐ Hogs/swine (3)
- ☐ Poultry (4)
- ☐ Sheep (5)
- ☐ Eggs (6)
- ☐ Apples (7)
- ☐ Alfalfa (8)
- ☐ Blueberries (9)
- ☐ Cabbage (10)
- ☐ Christmas Trees (11)
- ☐ Corn, popcorn (12)
- ☐ Corn, field corn (13)
- ☐ Corn, seed corn (14)
- ☐ Corn, sweet corn (15)
- ☐ Cotton (16)
- ☐ Cucumbers (17)
- ☐ Grapes (18)

- [ ] Green peppers (19)
- [ ] Hay (20)
- [ ] Oats (21)
- [ ] Peaches (22)
- [ ] Peanuts (23)
- [ ] Potatoes (24)
- [ ] Snapbeans (25)
- [ ] Sorghum (26)
- [ ] Soybeans (27)
- [ ] Strawberries (28)
- [ ] Sweet potatoes (29)
- [ ] Tomatoes (30)
- [ ] Tobacco (31)
- [ ] Watermelon (32)
- [ ] Wheat (33)
- [ ] Other fruit (34)
- [ ] Other vegetables (35)
- [ ] Other grains (36)
- [ ] Other farm animals (37)

Display This Question:

If Do you own or work on a farm? = No

Q4.5 Is your home located within 1 mile of a current or former crop or livestock farm?

- Yes (1)
- No (2)
- Don't know (3)

Display This Question:

If Do you own or work on a farm? = Yes

Or Is your home located within 1 mile of a current or former crop or livestock farm? = Yes

Q4.6 Approximately how far is your well from livestock waste storage (animal waste lagoon, pits, feedlots, etc)?

- Less than 50 feet (1)
- 50 - 100 feet (4)
- 100 - 200 feet (5)
- Greater than 200 feet (6)
- Don't know (3)

Q4.7 Is your home located within 5 miles of the following?

- Incinerator or coal burning power plant (1)
- Nuclear power plant (2)
- Industrial facility / manufacturing plant (3)
- Fertilizer Co-op (5)
- Don't know (4)

End of Block: Area

Start of Block: Diet

Q5.1 The following questions ask about specific foods you eat and drink.

Q5.2 During the **past 30 days**, on how many days did you have at least one glass of grape juice, pear juice or apple juice/cider?

- ○ 0 days (1)
- ○ 1 to 2 days (2)
- ○ 3 to 5 days (3)
- ○ 6 to 9 days (4)
- ○ 10 to 19 days (5)
- ○ 20 or more days (6)
- ○ Don't know (7)

Q5.3 During the **past 30 days,** on how many days did you consume rice-based foods or beverages? (for example, white rice, brown rice, rice cakes, rice crackers, rice bread, rice milk, rice water, horchata, etc)

- ○ 0 days (1)
- ○ 1 to 2 days (2)
- ○ 3 to 5 days (3)
- ○ 6 to 9 days (4)
- ○ 10 to 19 days (5)
- ○ 20 or more days (6)
- ○ Don't know (7)

Q5.4 During the **past 30 days**, on how many days did you consume seafood, including fish, smelts, shellfish, or seaweed? (for example, sandwiches, soups, salads, sushi, or canned or packaged tuna)

- 0 days (1)
- 1 to 2 days (2)
- 3 to 5 days (3)
- 6 to 9 days (4)
- 10 to 19 days (5)
- 20 or more days (6)
- Don't know (7)

Display This Question:

If During the past 30 days, on how many days did you consume seafood, including fish, smelts, shellf... = 1 to 2 days

Or During the past 30 days, on how many days did you consume seafood, including fish, smelts, shellf... = 3 to 5 days

Or During the past 30 days, on how many days did you consume seafood, including fish, smelts, shellf... = 6 to 9 days

Or During the past 30 days, on how many days did you consume seafood, including fish, smelts, shellf... = 10 to 19 days

Or During the past 30 days, on how many days did you consume seafood, including fish, smelts, shellf... = 20 or more days

Q5.5 Were any of the fish you consumed caught in Iowa lakes, ponds or rivers?

- Yes (1)
- No (2)
- Don't know (3)

End of Block: Diet

Start of Block: Health

Q6.1 The following questions are about your overall health.

Q6.2 What is your height?

- Less than 4' 6" (1)
- 4' 7" (2)
- 4' 8" (3)
- 4' 9" (4)
- 4' 10" (5)
- 4' 11" (6)
- 5' 0" (7)
- 5' 1" (8)
- 5' 2" (9)
- 5' 3" (10)
- 5' 4" (11)
- 5' 6" (12)
- 5' 7" (13)
- 5' 8" (14)
- 5' 9" (15)
- 5' 10" (16)
- 5' 11" (17)
- 6' 0" (18)
- 6' 1" (19)
- 6' 2" (20)
- 6' 3" (21)

○ 6' 4" (22)

○ 6' 5" (23)

○ 6' 6" or taller (24)

Q6.3 What is your weight?

○ Weight in pounds (1) ____

Display This Question:

If What is your sex? = Female

Q6.4 Are you currently pregnant?

○ Yes (1)

○ No (2)

○ Don't know (3)

Q6.5 During the **past 30 days**, did you take any Ayurveda, homeopathy, herbal medicines or spiritual remedies? (These products are not prescribed by medical professionals)

○ Yes (1)

○ No (2)

○ Don't know (3)

Display This Question:

If During the past 30 days, did you take any Ayurveda, homeopathy, herbal medicines or spiritual rem... = Yes

Q6.6 During the **past 5 days**, including today, on how many days did consume these products?

- ◯ 0 (1)
- ◯ 1 (2)
- ◯ 2 (3)
- ◯ 3 (4)
- ◯ 4 (5)
- ◯ All 5 days (6)

Q6.7 Have you ever been told by a doctor or other health professional that you had any of the following (check all that apply)

- ☐ Diabetes or sugar diabetes (other than during pregnancy) (1)
- ☐ High blood pressure (2)
- ☐ Heart disease (3)
- ☐ Lung disease (asthma, emphysema, COPD, etc) (4)
- ☐ Liver disease (5)
- ☐ None of these (6)

Q6.8 Have you ever been told by a doctor or other health professional that you had weak or failing kidneys? (Do not include kidney stones, bladder infections, or incontinence)

- ◯ Yes (1)
- ◯ No (2)

Q6.9 In the past 12 months, have you received dialysis (either hemodialysis or peritoneal dialysis)?

- ◯ Yes (1)
- ◯ No (2)

Q6.10 Have you ever been told by a doctor or other health professional that you had cancer or a malignancy?

- ◯ Yes (1)
- ◯ No (2)

Display This Question:

If Have you ever been told by a doctor or other health professional that you had cancer or a maligna... = Yes

Q6.11 What type(s) of cancer? (check all that apply)

- ▢ Bladder (1)
- ▢ Blood (2)
- ▢ Bone (3)
- ▢ Brain (4)
- ▢ Breast (5)
- ▢ Cervix (6)
- ▢ Colon (7)
- ▢ Esophagus (8)
- ▢ Gallbladder (9)
- ▢ Kidney (10)
- ▢ Lanynx/windpipe (11)
- ▢ Leukemia (12)
- ▢ Liver (13)
- ▢ Lung (14)
- ▢ Lymphoma (15)
- ▢ Melanoma (16)
- ▢ Mouth/tongue/lip (17)
- ▢ Ovary (18)

- [ ] Pancreas (19)
- [ ] Prostate (20)
- [ ] Rectum (21)
- [ ] Skin (non-melanoma) (22)
- [ ] Soft tissue (muscle or fat) (23)
- [ ] Stomach (24)
- [ ] Testis (25)
- [ ] Throat/phaynx (26)
- [ ] Thyroid (27)
- [ ] Uterus (28)
- [ ] Other (29) ____
- [ ] Don't know (30)

End of Block: Health

Start of Block: Occupation

Q7.1 This part of the survey will ask about your work experience.

Q7.2 Have you ever served on active duty in the U.S. Armed Forces, military Reserves, or National Guard? (Active duty does not include training for the Reserves or National Guard, but does include activation, for service in the U.S. or in a foreign country, in support of military or humanitarian operations.)

- ○ Yes (1)
- ○ No (2)

Q7.3 Which of the following were you doing **last week**?

- Working at a job or business (1)
- With a job or business but not at work (2)
- Looking for work (3)
- Not working at a job or business (4)

Display This Question:

If Which of the following were you doing last week? = With a job or business but not at work

Or Which of the following were you doing last week? = Looking for work

Or Which of the following were you doing last week? = Not working at a job or business

Q7.4 What is the main reason you did not work **last week**?

- Paid time off (vacation or sick leave) (1)
- Extended leave of absence (8)
- Going to school (2)
- Retired (3)
- Let go or fired from work (5)
- Disabled (6)
- Other (7) ____

Q7.5 Which of the following industries most closely matches the one in which you are currently, or were most recently, employed?

- ○ Agriculture (1)
- ○ Forestry, Fishing and Hunting (2)
- ○ Real estate or rental and leasing (3)
- ○ Mining (4)
- ○ Professional, scientific or technical services (5)
- ○ Utilities (6)
- ○ Management of companies or enterprises (7)
- ○ Construction/plumbing (8)
- ○ Administration or support services (9)
- ○ Waste management services (10)
- ○ Manufacturing/production (11)
- ○ Educational services (12)
- ○ Wholesale (13)
- ○ Healthcare or dentistry (14)
- ○ Social assistance (15)
- ○ Retail (16)
- ○ Arts, entertainment or recreation (17)
- ○ Transportation or warehousing (18)
- ○ Accommodation or food service (19)
- ○ Information (20)
- ○ Oil or gas (21)

- ○ Other services (except public administration) (22)
- ○ Finance or insurance (23)
- ○ Other (24) ____

Q7.6 Which of the following best describes your **occupation (or role)** within this industry?

- Management (1)
- Business and Financial Operations (2)
- Computer and Mathematical (3)
- Architecture and Engineering (4)
- Life, Physical, and Social Science (5)
- Community and Social Service (6)
- Legal (7)
- Educational Instruction and Library (8)
- Arts, Design, Entertainment, Sports, and Media (9)
- Healthcare Practitioners and Technical (10)
- Healthcare Support (11)
- Protective Service (12)
- Food Preparation and Serving Related (13)
- Building and Grounds Cleaning and Maintenance (14)
- Personal Care and Service (15)
- Sales and Related (16)
- Office and Administrative Support (17)
- Farming, Fishing, and Forestry (18)
- Construction and Extraction (19)
- Installation, Maintenance, and Repair (20)
- Production (21)

- Transportation and Material Moving Occupations (22)
- Academic Research (23)
- Student (24)
- Other (25)
- Retired (26)

Q7.7 Which of the following categories best describes your total household income in the 12 months, including wages, salaries, Social Security or retirement benefits, help from relatives and so forth?

- Less than $25,000 (1)
- $25,000 - 49,999 (2)
- $50,000 - 74,999 (3)
- $75,000 - 99,999 (4)
- $100,000 - 149,999 (5)
- $150,000 or more (6)

End of Block: Occupation

Start of Block: Pesticides

Q8.1 This part of the survey will ask about your use of chemicals to control weeds and insects.

Display This Question:

If Do you own or work on a farm? = Yes

Or Which of the following industries most closely matches the one in which you are currently, or wer... = Agriculture

Or Which of the following best describes your occupation (or role) within this industry? = Farming, Fishing, and Forestry

Q8.2 Within the past 12 months, have you personally mixed, loaded, or applied any pesticides on crops (NOT including home garden use)?

- Yes (1)
- No (2)

Display This Question:

If Within the past 12 months, have you personally mixed, loaded, or applied any pesticides on crops... = Yes

Q8.3 Which of the following have you used? Select all that apply.

- ▢ Fertilizers (chemicals that are added to the soil to support plant growth) (1)
- ▢ Herbicides (chemicals that are used to destroy unwanted vegetation) (2)
- ▢ Rodenticides (chemicals that are used to kill rodents or small mammals) (3)
- ▢ Insecticides (chemicals that are used to kill insects) (4)
- ▢ Fungicides (chemicals that are used to kill fungi or their spores) (5)
- ▢ Don’t know (6)

Q8.4 In the **past 5 days**, including today, were any chemical products applied to your lawn or garden to kill weeds?

- ◯ Yes (1)
- ◯ No (2)
- ◯ Don't know (3)

Display This Question:

If In the past 5 days, including today, were any chemical products applied to your lawn or garden to... = Yes

Q8.5 If you know the name of the weed control product, please type it here. Otherwise, skip to the next question.

____

Q8.6 In the past 5 days, including today, were any chemical products applied in or around your home to control fleas, roaches, ants, termites or other insects (including on household pets)?

- ◯ Yes (1)
- ◯ No (2)
- ◯ Don't know (3)

Display This Question:

If In the past 5 days, including today, were any chemical products applied in or around your home to... = Yes

Q8.7 If you know the name of the insect control product you used, please type it here. Otherwise skip to the next question.

____

End of Block: Pesticides

Start of Block: Personal Care

Q9.1 This section of the survey will ask about your use of your personal care products.

Q9.2 During the past 5 days, including today, on how many days did you use **facial cosmetics**, including foundation, make-up primer, concealer, powder, setting spray, lipstick/lip tint, eyeliner, eyeshadow, mascara, or blush?

- 0 (1)
- 1 (2)
- 2 (3)
- 3 (4)
- 4 (5)
- All 5 days (6)

Q9.3 During the past 5 days, including today, on how many days did you use **hair cosmetics**, including hair spray, hair shine, styling gel, mousse/foam, curl cream, leave-in conditioner, pomade/grease/wax, hair glue (for wigs, extensions or weaves), or detangler?

- 0 (1)
- 1 (2)
- 2 (3)
- 3 (4)
- 4 (5)
- All 5 days (6)

Q9.4 During the past 5 days, including today, on how many days did you use **personal hygiene products**, including toothpaste, mouthwash, deodorant/antiperspirant, perfume/cologne/body spray, sunscreen, face cream/moisturizers, face mask, hand or body lotions, body oil, hand soap, body soap/shower gel, facial soap/cleanser, shampoo, or conditioner?

- 0 (1)
- 1 (2)
- 2 (3)
- 3 (4)
- 4 (5)
- All 5 days (6)

Display This Question:

If What is your sex? = Female

Q9.5 During the past 5 days, including today, on how many days did you use **genital hygiene products**, including tampons, sanitary napkins/pads, douche, feminine spray, vaginal deodorant suppositories, vaginal wipes/towelettes, vaginal sashes/cleansers, anti-itch cream, lubricant, or shaving cream?

- 0 (1)
- 1 (2)
- 2 (3)
- 3 (4)
- 4 (5)
- All 5 days (6)

Q9.6 During the past 30 days did you use any of the following products? (select all that apply)

- ▢ Nail polish, gel, acrylic or wraps (1)
- ▢ Hair dye (2)
- ▢ Hair perm (3)
- ▢ Hair relaxer (straightener) (4)
- ▢ Skin lighteners (5)
- ▢ None of these (6)

End of Block: Personal Care

Start of Block: Tobacco

Q10.1 This final section asks about your use of cigarettes and other tobacco products. Do not include marijuana.

Q10.2 Have you smoked more than 100 cigarettes in **your entire life** (1 pack = 20 cigarettes)?

- ○ Yes (1)
- ○ No (2)

Skip To: Q10.6 If Have you smoked more than 100 cigarettes in your entire life (1 pack = 20 cigarettes)? = No

Q10.3 During the **past 30 days**, on how many days did you smoke cigarettes?

- 0 days (1)
- 1 to 2 days (2)
- 3 to 5 days (3)
- 6 to 9 days (4)
- 10 to 19 days (5)
- 20 or more days (6)
- I have quit (7)

Display This Question:

If During the past 30 days, on how many days did you smoke cigarettes? = I have quit

Q10.4 About how long has it been since you quit smoking cigarettes?

- Less than 1 month (1)
- 1 to 2 months (2)
- 3 to 6 months (3)
- 7 to 12 months (4)
- More than 1 year (5)

Display This Question:

If During the past 30 days, on how many days did you smoke cigarettes? = 1 to 2 days

Or During the past 30 days, on how many days did you smoke cigarettes? = 3 to 5 days

Or During the past 30 days, on how many days did you smoke cigarettes? = 6 to 9 days

Or During the past 30 days, on how many days did you smoke cigarettes? = 10 to 19 days

Or During the past 30 days, on how many days did you smoke cigarettes? = 20 or more days

Q10.5 During the **past 30 days**, on the days that you smoked, how many cigarettes did you smoke per day (1 pack = 20 cigarettes)?

- Enter Number (1) ____

Q10.6 During the **past 5 days**, including today, did you use tobacco products other than cigarettes, including pipes, cigars, cigarillos (little cigars), water pipes, hookahs, e-cigarettes (also known as vape pens, hookah pens, e-hookahs or e-vaporizers), or smokeless tobacco (chew, snus, of snuff)?

- ○ Yes (1)
- ○ No (2)

Q10.7 Does anyone else who lives in the household smoke tobacco products?

- ○ Yes, inside the home (1)
- ○ Yes, outside the home (2)
- ○ No (3)

End of Block: Tobacco
