# Supplementary material for: Human biomonitoring without in-person interaction: public health engagements during the COVID-19 pandemic and future implications
Source: BMC Med Res Methodol. 2024 Feb 28;24:53. doi: 10.1186/s12874-024-02165-x (PMC10900566; doi:10.1186/s12874-024-02165-x)
Supplement: Supplementary file 3 — Supplementary Material 3 [file 12874_2024_2165_MOESM3_ESM.pdf]

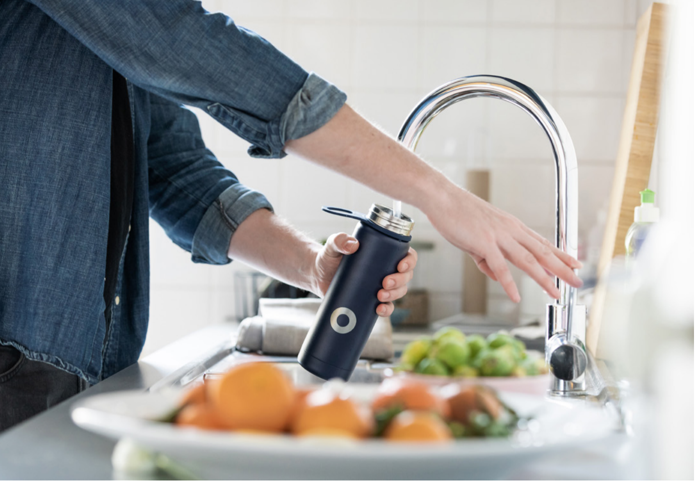

# THANK YOU

FOR PARTICIPATING IN THE
IOWA BIOMONITORING PROGRAM

## Before you begin:

- Pay close attention to how your kit was packed upon arrival. When shipping samples back to the lab, pack the cooler in the same way.
- Freeze the provided ice packs.
- Plan to complete all tasks in one day (about 1 hour).
- Follow the enclosed instructions carefully and in order. Visit our website for more information about collecting and shipping samples.
- Go to https://www.ups.com/dropoff/ or call (800) 742-5877 and say "drop off locations" to find your nearest UPS drop off location.

## When you are done:

The Iowa Biomonitoring Program will mail you a $15 gift card following submission of the questionnaire and a $35 gift card when all samples are received.

Water results as well as results for arsenic, cadmium, lead, and uranium in urine will be mailed when testing is complete. This will take several months.

# ABOUT BIOMONITORING

We are exposed to many chemicals in our everyday environment. Environmental chemicals enter our bodies through air, water, food, soil, dust, and commercial products. Many chemicals are harmless, but some may be harmful to our health. Through biomonitoring, scientists study which chemicals people may be exposed to and the amount of those chemicals that get into their bodies, usually by testing human samples such as urine. This important work helps us understand how people may be exposed to potentially harmful chemicals and find ways to reduce exposures.

By participating in this surveillance program, you are helping scientists better understand certain chemical exposures that may occur in Iowa.

The Iowa Biomonitoring Program is a public health surveillance project conducted by the State Hygienic Laboratory at the University of Iowa and is funded by the Centers for Disease Control and Prevention.

## LEARN MORE

### ASK QUESTIONS

shl-biomonitoring@uiowa.edu
319-467-4503

### VISIT US ONLINE

www.biomonitoring.shl.uiowa.edu

IOWA
State Hygienic Laboratory

IOWA

# IOWA BIOMONITORING PROGRAM

## Sampling Kit

State Hygienic Laboratory

Iowa's Environmental and Public Health Laboratory

**STEP 1**

# URINE SAMPLE

## First morning sample

***NOTE: Collect the urine sample immediately upon waking for the day.***

1. Label the container.
2. Wash your hands with soap and water.
3. Separate the skin folds around the urinary opening.
4. Using the provided towelette packets, clean the urinary opening and surrounding areas from front to back.
5. Urinate into the toilet for a few seconds and then stop.
6. Place the empty container into the path of the stream.
7. Restart the urine stream and collect into the container.
8. Tightly screw the cap onto the container.
9. Wash your hands with soap and water.
10. Place the container and absorbent pad into the biohazard bag.
11. Freeze the packaged sample until you are ready to ship it back to the laboratory.
12. Prior to shipping, place the sample in the small Styrofoam case and secure it closed with the rubber band.

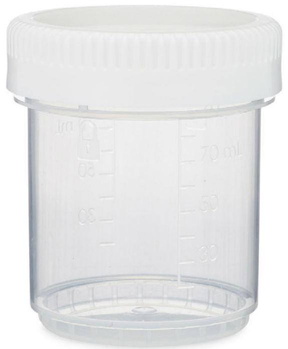

**STEP 2**

# WATER SAMPLE

## Indoor drinking water sample

*CAUTION: All bottles are sterile. Plastic bottles contain chemical preservatives. Do not overfill the bottles or touch inside the bottles or lids. In case of contact with eyes, skin, or clothes, immediately rinse with water for up to 15 minutes and call a physician if irritation persists. We recommend wearing the provided disposable vinyl gloves. Protective eye-wear (not provided) is also advised.*

1. Select the sink you most often use for drinking and cooking. Set a clean paper towel on the counter.
2. Label each bottle with the date, time, and participant ID.
3. Wash your hands with soap and water. Put on gloves.
4. Turn the cold water on high for 5 seconds, then reduce the water to a slow, steady stream (pencil width).
5. One bottle at a time:
   - remove the lid from the bottle (being careful not to touch inside the lid or containers)
   - place the lid on the clean paper towel
   - slowly fill the bottle to the shoulder, 1/2 inch below the lid (do not overfill the bottle or pour water out)
   - tightly screw the lid back onto the bottle
   - repeat until each of the 6 bottles are filled
6. Immediately refrigerate the samples until you are ready to ship them back to the laboratory (within 3 days).

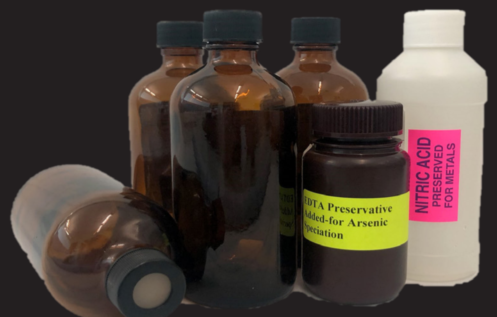


**STEP 3**

# QUESTIONNAIRE

Access the questionnaire online at:

https://redcap.icts.uiowa.edu/redcap/surveys/

- Enter your unique Access Code in the enclosed cover letter.
- Please complete the survey in one sitting so progress is not lost. The survey should take around 25 minutes.

***NOTE: Some internet browsers fail to access the online questionnaire. For best results, try Firefox, Microsoft Edge, or Google Chrome. Need help? Contact us! We can provide a link or PDF by email or mail a hard copy.***

**STEP 4**

# RETURN SAMPLES

- Ship samples within 3 days of collection. Follow the cooling instructions (at left) for each set of samples until they are shipped.
- Complete the top portion of the 'Sample Collection Form'. Do not fill out the bottom 'chain of custody' section.
- The cooler should include:
  - All sample bottles with completed labels
  - Frozen ice packs
  - Completed paperwork sealed in the plastic zip bag
- Fill all gaps with bubble wrap to prevent breakage.
- Place the cooler into the box and securely tape it shut.
- Place the return shipping label and "Exempt Human Specimen" sticker on the outside of the box.
- Go to https://www.ups.com/dropoff/ or call (800) 742-5877 and say "drop off locations" to find your nearest UPS drop off location.
