# Supplementary material for: Human biomonitoring without in-person interaction: public health engagements during the COVID-19 pandemic and future implications
Source: BMC Med Res Methodol. 2024 Feb 28;24:53. doi: 10.1186/s12874-024-02165-x (PMC10900566; doi:10.1186/s12874-024-02165-x)
Supplement: Supplementary file 4 — Supplementary Material 4 [file 12874_2024_2165_MOESM4_ESM.pdf]

Iowa Biomonitoring Program: MAKE A PLAN

1 YOUR KIT ARRIVED!	2 URINE SAMPLES	3 WATER SAMPLES	4 QUESTIONNAIRE	5 SHIP TO LAB
❑ Freeze ice packs<br>❑ Keep all packaging	❑ Collect<br>❑ Freeze	❑ Collect<br>❑ Refrigerate	❑ Complete online	❑ Ship within 3 days

NOTE: **Steps 2-4** should all occur on the **same day**.

For more information, visit our website at **biomonitoring.shl.uiowa.edu** and see the enclosed instructional pamphlet for additional details.
