# Supplementary material for: Human biomonitoring without in-person interaction: public health engagements during the COVID-19 pandemic and future implications
Source: BMC Med Res Methodol. 2024 Feb 28;24:53. doi: 10.1186/s12874-024-02165-x (PMC10900566; doi:10.1186/s12874-024-02165-x)
Supplement: Supplementary file 5 — Supplementary Material 5 [file 12874_2024_2165_MOESM5_ESM.pdf]

ENROLL TODAY

➔ HTTPS://REDCAP.ICTS.UIOWA.EDU/REDCAP/SURVEYS/

access code sticker

IOWA

State Hygienic Laboratory

BIOMONITORING: WHY IT MATTERS

Helps Iowa assess well water contaminants

Measures levels of certain chemicals in residents

Could identify vulnerable communities

May improve knowledge about exposures and human health

Will inform environmental policy to reduce exposure

→ BIOMONITORING.SHL.UIOWA.EDU
